# Constructing the context through goals and schemata: top-down processes in comprehension and beyond

**DOI:** 10.3389/fpsyg.2015.00651

**Published:** 2015-05-19

**Authors:** Marco Mazzone

**Affiliations:** Department of Humanities, University of Catania, Catania, Italy

**Keywords:** schemas, context, mindreading, hierarchical representation, pragmatics

## Abstract

My main purpose here is to provide an account of context selection in utterance understanding in terms of the role played by schemata and goals in top-down processing. The general idea is that information is organized hierarchically, with items iteratively organized in chunks—here called “schemata”—at multiple levels, so that the activation of any items spreads to schemata that are the most accessible due to previous experience. The activation of a schema, in turn, activates its other components, so as to predict a likely context for the original item. Since each input activates its own schemata, conflicting schemata compete with (and inhibit) each other, while multiple activations of a schema raise its likelihood to win the competition. There is therefore a double movement—with bottom-up activation of schemata enabling top-down prediction of other contextual components—triggered by multiple sources. Another claim of the paper is that goals are represented by schemata placed at the highest-levels of the executive hierarchy, in accordance with Fuster’s model of the brain as a hierarchically organized perception-action cycle. This account can be considered, in part at least, a development of ideas contained in Relevance Theory, though it may imply that some other claims of the theory are in need of revision. Therefore, a secondary purpose of the paper is a contribution to the analysis of that theory.

## Introduction

The problem of adequately accounting for the cognitive role of context does not affect only pragmatics: most, and possibly all, human behaviors require taking into account indefinitely changeable contexts and even deciding what counts as the relevant context in the present case. As a matter of fact, one of the most developed theories in cognitive pragmatics is Relevance Theory (from now on, RT; Sperber and Wilson, 1986/1995), in which communication is analyzed as a special case of cognition precisely because both cognition in general and communication in particular have the problem of selecting what is relevant in the present context (or which is the presently relevant context).

My main purpose here is to provide an account of context selection in utterance understanding in terms of the role played by schemata and goals in top-down processing. This account can be considered, in part at least, a development of ideas contained in RT, though it may imply that some other claims of the theory are in need of revision. Therefore, a secondary purpose of the paper is a contribution to the analysis of RT. On the other hand, I also aim to show that the proposed account, which is based on a quite general mechanism, is consistent with explanations of flexible behavior in linguistics, in theory of concepts, and in psychological, neuroscientific, and computational theories of action control.

Relevance Theory has conceived of utterance interpretation as a special case of the search for relevance in cognition. Utterances raise expectations of relevance in the addressees, thus triggering a search for contexts in which they are actually made relevant. In practice, non-demonstrative inferences are constructed, with encoded meaning and contextual assumptions acting as premises that license contextual conclusions.^1^ Utterance interpretation thus amounts to identifying the relevant cognitive context, that is, the appropriate and intended set of contextual assumptions (and conclusions). In this account, an important role is played by the organization of memory, more precisely by the differential accessibility of contents: these can be more or less strongly associated to (and then more or less easily activated by) the inputs to be processed. Relevance theorists have occasionally noted that such differential accessibility may depend on the fact that memory is organized in chunks, a point that notions such as schemata, frames, scripts etc. are intended to account for.

In this paper, I take this idea very seriously and attempt to frame it within a general model of the human brain architecture and cognitive processing. This model, proposed by Fuster (2001, 2003, 2014), conceives our cortex as organized along two highly interconnected hierarchies of representations, the sensory and the motor one, which together constitute a perception-action cycle. The representations are hierarchically organized in the sense that higher cortical layers provide the structure by which items at lower levels are arranged together, which is a different way to say that items are iteratively organized in chunks at multiple levels. I will call “schemata” the higher-level representations describing the organization of items at lower levels.

The general idea I will pursue is the following.^2^ The activation of items at each level gives access in a probabilistic manner to schemata they pertain to, that is, activation spreads to schemata that are the most accessible due to previous experience. The activation of a schema, in turn, activates its other components, so as to predict a likely context for the original item. However, such prediction can be either confirmed or refuted by the actual context—more precisely, by the variety of the current inputs each of which activates its own schemata, and therefore its own predictions about context. Conflicting schemata compete with (and inhibit) each other, while multiple activations of a schema raise its likelihood to win the competition. There is therefore a double movement—with bottom-up activation of schemata enabling top-down prediction of other contextual components—triggered by multiple sources.

In utterance understanding, this picture applies both to linguistic and non-linguistic inputs. Each of them spreads activation to schemata, thus providing probabilistic predictions about their possible context. Since each input acts as context for the others, those predictions are in fact assessed against each other.

Another crucial assumption of this paper is that goals are represented by schemata placed at the highest-levels of the executive hierarchy, that is, at the top of the motor stream of the perception-action cycle described by Fuster. In particular, the most abstract goals are located within the prefrontal cortex (PFC), which is responsible for controlled action, that is, for top-down control of action in a processual sense. I will shortly examine how these different senses of “top-down” are related with each other and actually involved in utterance understanding: not only do linguistic and non-linguistic inputs activate schemata in general, they also activate schemata specifically representing goals, and this activation may result in attentive (PFC-driven) processing of utterances.

In line with Grice, but also with suggestions coming from Levinson and from RT, I will propose in fact that utterance interpretation requires forming hypotheses about goals/intentions. On the one hand, utterances are evidence provided by the speaker to the addressee in order for her to recognize a communicative intention that may go far beyond coded meaning. On the other hand, recognition of this intention requires its being placed within an entire system of goals, since communicative intentions are in general means for other goals, which can or cannot be themselves communicative. In a sense, then, the purpose of communication is the shared representation of a set of (communicative and non-communicative) goals by the speaker and the addressee.

In this perspective, language production and understanding appear to be just components of a more general top-down/bottom-up cortical dynamic involved in the execution and understanding of intentional action. However, such action-oriented view of language is not uncontroversial, and I will discuss some theses of RT that might turn out to be in conflict with it.

My discussion of RT requires an important qualification. What I propose here, apart from being a development of ideas put forth by RT, can be interpreted in part as an attempt to specify how RT might be implemented at the neuro-computational level. In practice, my account of the associative dynamic by which inputs activate a variety of schemata that compete with, or strengthen the activation of, each other, may provide a unitary explanation of the neuromechanics of a range of phenomena spanning different levels of linguistic processing. To this extent, I see my proposal as largely compatible with RT.^3^ The only problem I raise here concerns a quite specific issue, that is, the role assigned to quantitative expectations of relevance. More specifically, I discuss the idea that in pragmatic interpretation there is an assessment of the *amount* of cognitive effects. As an alternative, I will consider a different route that has been explored by RT, based on the idea of expectations about specific *types* of cognitive effects. As I will argue, while the idea of a quantitative assessment of cognitive effects is consistent with the view of communication as geared to maximization of information, the alternative proposal is more compatible with the view of communication as based on the variety of human purposes.

## RT and Pragmatic Context

### Context and Relevance

First of all, let us consider in some detail the crucial role that RT assigns to context, to the point that constructing the right context comes to be seen as the main part of the entire process of utterance interpretation. Some terminological clarifications are in order. What RT is in fact concerned with is the cognitive context, that is, the set of assumptions needed in order for the addressee to infer the intended conclusions from the coded meaning of utterances.^4^ This notion has then to be distinguished from the more standard notion of linguistic and situational context, that is, the factual linguistic and extra-linguistic environment in which the utterance is embedded and which provides further inputs to cognitive processes. Those inputs contribute to activate the assumptions involved in the interpretation of coded meaning: that is, the *factual* context contributes to the activation of the *cognitive* context.

In this perspective, the context is not something given before the interpretation starts, contrary to what has often been assumed:

In much of the pragmatic literature, events are assumed to take place in the following order: first the context is determined, then the interpretation process takes place, then relevance is assessed. […RT] suggests a complete reversal of the order of events in comprehension. It is not that first the context is determined, and then relevance is assessed. On the contrary, people hope that the assumption being processed is relevant (or else they would not bother to process it at all), and they try to select a context which will justify that hope: a context which will maximize relevance (Sperber and Wilson, 1986/1995, p 141–142).

Thus, the interpretation process is not preceded by the selection of a context, rather the latter is constitutive of the former, and this process in turn does not precede relevance assessment, on the contrary it is driven from the beginning by expectations of relevance. Such expectations are embodied, so to speak, in the mechanism by which interpretation is performed, insofar as this mechanism works so as to ensure that a relevant context is selected. In this sense, RT claims that cognition in general, and utterance interpretation as a special case, is geared to maximization of relevance.

What then is relevance, and by which mechanism is it attained in utterance understanding? According to RT, intuitively an input is relevant when its processing yields a positive cognitive effect, specifically it “is relevant to an individual when it connects with background information he has available to yield conclusions that matter to him” (Wilson and Sperber, 2002a, p. 251). However, in a realistic account cognitive effects must be balanced against the cognitive effort required in order to get them. Therefore, a complete definition of relevance has two sides, a positive and a negative one:

a. Other things being equal, the greater the positive cognitive effects achieved by processing an input, the greater the relevance of the input to the individual at that time.
b. Other things being equal, the greater the processing effort expended, the lower the relevance of the input to the individual at that time (Wilson and Sperber, 2002a, p. 252–253).

When utterance interpretation is at issue, this definition is intended to refer to relevance of interpretations (vs. inputs). The mechanism by which interpretations that are relevant in this sense are construed is described as a heuristic in two steps, the “relevance-theoretic comprehension procedure”:

a. Follow a path of least effort in computing cognitive effects: Test interpretive hypotheses […] in order of accessibility.
b. Stop when your expectations of relevance are satisfied (Wilson and Sperber, 2002a, p. 260).

The first step of the comprehension procedure is easily understood in the following terms. An interpretation is built following a path of least effort, that is, contextual assumptions licensing contextual conclusions (in relation to the coded meaning) are selected in order of accessibility. This step does not require more than a simple associative mechanism, which makes some assumptions more accessible than others given the utterance and the factual context. As to the second step, it prescribes some sort of assessment of the obtained interpretation against previous expectations of relevance. However, I see here a potential problem, which has consequences for the proposed definition of relevance as well. RT has provided only vague suggestions about how this assessment might be performed, as Sperber and Wilson (1987, p. 742) themselves admit:

Relevance, as it affects cognition, is not computed or numerically measured but monitored or assessed, yielding only gross absolute judgments and, in certain types of cases only, finer relative judgments. Suppose that the brain is sensitive to the amount of reorganization brought about by the processing of some information and to the expenditure of energy thus incurred, just as it is sensitive to changes of posture and expenditure of energy in the case of bodily movement. This is very vague—hopelessly so, some AI [artificial intelligence] people may think—but it is not so vague that it could not be false, and it is what we are claiming anyhow.

Starting from Sperber and Wilson (1986/1995, p. 130), relevance theorists have occasionally repeated without further development such “speculation,” as they call it, according to which “contextual effects and mental effort, just like bodily movements and muscular effort, must cause some symptomatic physico-chemical changes.” To my knowledge, none of the supporters of RT has ever tried to relate this speculation to any known cognitive mechanism. However, I want to show that RT has provided a number of clues pointing toward a different direction.

To start with, it should be noted that, while this speculation concerns the assessment of both effects and effort, there is in fact an asymmetry between them in the comprehension procedure. The minimization of effort is apparently ensured already by the first step, that is, by accessing the most accessible (i.e., the least costly) interpretations in the first place. This may suggest that what needs to be further assessed, as required by the second step, is (the maximization of) cognitive effects. As a matter of fact, relevance theorists often refer to expectations of relevance specifically in terms of expectations about the amount of cognitive effects. In sum, while the criterion of effort can be accounted for very naturally in terms of associative accessibility, it is the criterion of cognitive effects that needs to make an appeal to the above speculation about “symptomatic physico-chemical changes.”

However, RT has also considered two alternative views about expectations of cognitive effects, even if only implicitly.

### Does Effort do Everything?

According to the first suggestion, the criterion of minimization of effort alone might be sufficient to drive the cognitive system toward the maximization of benefits. This is suggested in passing by Sperber and Wilson (1996), in a passage where effort is first considered, as one would expect, as the purely negative side of relevance, but then an unexpected question follows (emphasized in italics):

when expectations of effect are wholly indeterminate, the mind should base itself on considerations of effort: pick up from the environment the most easily attended stimulus, and process it in the context of what comes most readily to mind. *Ceteris paribus*, what is easier is more relevant, if it is relevant at all. *But what are the chances that what comes more easily to mind is, in fact, relevant?* [emphasis mine] They would be close to nil, if saliency in the environment and accessibility in memory were both random, and moreover uncorrelated (Sperber and Wilson, 1996).

The question is unexpected, because there seems to be no reason why “what comes more easily to mind” should be relevant over and beyond the fact that it is, *ceteris paribus*, relevant *by definition* simply because it demands little effort (negative side of relevance). Of course, easily accessed stimuli (and interpretations) might happen to be almost entirely irrelevant on the positive side, that is, they might have little or no cognitive effects. But it is precisely in order to avoid that risk that the second step of the comprehension procedure is required, while here Sperber and Wilson seem to wonder whether ease of access can *by itself* ensure some relevance on the positive side. And in fact the previous quotation is followed by an evolutionary argument to the effect that what requires little cognitive effort is also likely to be, so to speak, the right sort of information, independently of any further mechanism for ensuring that sufficient cognitive effects are attained:

But humans are evolved organisms with learning capacities of sorts, so it is not too surprising to find that they spontaneously pay more attention […] to objects and events that, on average, are more likely to be relevant to them.

For the same reason, it is not surprising that the perceptual categorization of a distal stimulus should tend to activate related information in memory. […] Nor is it surprising that memory is so organized that pieces of information that are likely to be simultaneously relevant tend to be co-accessed or co-activated in chunks variously described in the literature as “concepts” “schemas,” “scripts,” “dossiers,” etc (Sperber and Wilson, 1996).

In practice, the suggestion is made that relevance on the negative side of the notion (the ease-of-access side) is sufficient to ensure relevance also on the positive side. More specifically, the organization of information in memory, by means of concepts, schemas etc., is suggested to ensure that ease of access of a given content is a reliable sign of its (probabilistic) contextual significance.

I want to emphasize that this suggestion is very close to a proposal made by Recanati (2004) with regard to what he calls “primary pragmatic processes.” These are processes by which the coded meaning of utterances is adjusted and expanded in order to get the contextually appropriate and complete proposition that is today called the “explicit meaning” of the utterance. In Recanati’s view, these processes, unlike the genuinely inferential ones required for deriving the implicit meaning of the utterance from its explicit meaning, are simple associative processes based on spreading of activation in conceptual networks. According to Recanati, this spreading activation is not wholly unconstrained and blind insofar as it activates schemata^5^, which ensure a search for coherent interpretations: “Coherent, schema-instantiating interpretations […] tend to be selected and preferred over non-integrated or “loose” interpretations” (Recanati, 2004, p. 37). This occurs because of a double associative dynamic: on the one hand, on the bottom-up direction of the dynamic, “a schema is activated by, or accessed through, an expression whose semantic value corresponds to an aspect of the schema”; on the other hand, on the top-down direction, the “schema thus activated in turn raises the accessibility of whatever possible semantic values for other constituents of the sentence happen to fit the schema” (Recanati, 2004, p. 37).^6^

Interestingly, not only have Sperber and Wilson (1996) suggested a similar role for schemata (scripts etc.) in the context of the theoretical discussion mentioned above; relevance theorists have also appealed to this mechanism in various analyses of concrete examples. For instance, let us consider Carston’s (2007) analysis of the following utterance:

(1) I’m going to the bank now to get some cash.

Since there are two possible meanings for “bank” (FINANCIAL INSTITUTION, RIVER SIDE), the problem is how the addressee may come to choose the right one. Carston (2007) makes the hypothesis that starting from the activation of CASH, a stereotypical frame or script for GETTING MONEY FROM A BANK_1_ (where BANK_1_ = FINANCIAL INSTITUTION) is recalled, thus strengthening the activation of BANK_1_. As in Recanati (2004), the idea is that something like a schema is activated bottom-up by some of its component (GETTING MONEY FROM A BANK_1_ is activated by the concept GETTING MONEY, which is activated in turn from the words “to get some cash”), and then it raises top-down the accessibility of its other components (BANK_1_ = FINANCIAL INSTITUTION), so that the concept FINANCIAL INSTITUTION comes to be preferred as the interpretation of “bank.”

In this example, the relevant meaning of “bank” can be selected by nothing else than ease of access, thanks to the fact that—in Sperber and Wilson’s (1996) words—“memory is so organized that pieces of information that are likely to be simultaneously relevant tend to be co-accessed or co-activated in chunks.”^7^

### Expectations about Either the Amount or the Type of Effects?

If our previous considerations are right, it might be the case that, contrary to the standard view in RT, no assessment of cognitive effects is required in addition to the negative criterion of accessibility. However, in many occasions relevance theorists have claimed instead that simple accessibility does not constrain interpretations enough, and that some independent assessment of cognitive benefits is needed. Specifically, the standard view is that interpretations must be assessed against some expected amount of cognitive effects. But relevance theorists have also explored a different route, that is, the idea that our expectations of relevance concern the type rather than the amount of cognitive benefits. I intend to argue, first, that there is a substantial difference in conceiving expectations of relevance in terms of the type vs. the amount of cognitive effects, and, second, that, at a closer analysis, this hypothesis points to the same direction as the suggestion that cognitive efforts may suffice to explain the search for relevance.

First of all, let us note that relevance theorists explicitly mention expectations about the *type* of cognitive effects, either with or without mention of their *amount*. For an example of the mention of both, consider this quotation from Carston (2007, p. 20, emphasis mine): “an utterance automatically triggers quite specific expectations of relevance in its addressee, that is, expectations concerning *both the quantity and the kind* of cognitive effects (implications) it will yield if optimally processed.” Mention of the type has become especially frequent in recent versions of RT, the ones characterized—in Wilson’s (2004, p. 352) words—by “the introduction of the mutual adjustment process (e.g., Sperber and Wilson, 1998; Wilson and Sperber, 2002b, 2004).” The idea is that pragmatic processing does not operate sequentially, by means of only forward inferences from the proposition expressed to the intended cognitive effects (passing through the selection of appropriate contextual assumptions). On the contrary, there is a parallel process based on both forward and backward inferences, in the course of which explicit content, contextual assumptions and cognitive effects are mutually adjusted to each other:

Mutual adjustment is seen as taking place in parallel rather than in sequence. The hearer does not first identify the proposition expressed, then access an appropriate set of contextual assumptions and then derive a set of cognitive effects. In many cases […], he is just as likely to reason backward *from an expected cognitive effect* to the context and content that would warrant it (Wilson, 2004, p. 353; emphasis mine).

As the last sentence suggests, the backward inferences involved in the mutual adjustment process require expectations about specific kinds of cognitive effects. For one example (from Wilson and Carston, 2007), consider the following exchange:

(2) Peter: Will Sally look after the children if we get ill?

Mary: Sally is an angel.

Apparently the implicit content conveyed by Mary’s utterance is an affirmative answer to the question raised by Peter, something like SALLY WILL LOOK AFTER THE CHILDREN IF WE GET ILL. This can be seen as the conclusion of an inference having as its premises the explicit content of Mary’s utterance and possibly some contextual assumptions. As to the explicit content, however, the concept that the word “angel” contributes to it cannot be the encoded concept ANGEL which has as its property SUPERNATURAL BEING OF A CERTAIN KIND. It must be instead a different concept obtained by adjusting the encoded concept to the context. A natural explanation of this adjustment is precisely by means of a backward inference from the expected conclusion. Since Peter’s question requires a yes/no answer, it can be thought to raise the expectation that Mary intends to claim either SALLY WILL LOOK AFTER THE CHILDREN IF WE GET ILL or its negation, and this expectation in turn licenses a backward inference toward the explicit content, which has to be coherent with either the affirmative or the negative claim. Thus, the concept ANGEL has to be adjusted until the explicit content provides a premise (for instance, SALLY IS KIND AND CARING) which has either the affirmative or the negative claim as its conclusion.

The example clearly shows how expectations about *specific cognitive effects* are involved in drawing backward inferences. This makes the notion of expected *type* (of cognitive effects) significantly different from the one of expected *amount*: while the former concerns specific contents that imposes backward constraints on the content of the premises, the latter is devoid of any content and therefore can at most permit a comparison with the amount of actual cognitive effects. Another key difference is that the notion of backward inferences from expected cognitive effects admits of a natural explanation in terms of ease of access via schemata. In our previous example, the expected cognitive effect that Mary intends to give a yes/no answer to Peter depends on a well-learned schema connecting yes/no questions and yes/no answers. Peter’s question is likely to activate this schema, which in turn activates the expectation about Mary’s possible answer. On the contrary, with regard to the assessment of the amount of cognitive effects, RT provides no better explanation than the vague speculation about “symptomatic physico-chemical changes.”

To summarize, we have described two alternatives to the standard RT’s claim that actual cognitive effects are assessed against expectations about their amount. Now it turns out that these alternatives are not only complementary but also explainable in terms of the same mechanism: ease of associative access and the schematic organization of memory (i.e., the organization of memory in “chunks”). In fact, expectations about specific kinds of cognitive effects apparently amount to associative activations of contextual conclusions via schemata. Thanks to this common mechanism, contextual assumptions and conclusions can be activated both by words constituting the utterance (via forward inferences) and by inputs from the linguistic and non-linguistic context (via backward inferences). In this perspective, instead of an assessment of the amount of cognitive effects against expectations of relevance, the process may be described as a mutual assessment of different predictions about the context. In other words, the suggestion is that hypotheses about the cognitive context are activated from different sources (utterance, linguistic and extra-linguistic context) and then assessed against each other, in a way that appeals only to ease of access (the negative side of relevance) and the organization of memory: hypotheses that are coherent with each other within the schematic organization of memory are activated more strongly and win the competition.

Let me shortly specify what this reconstruction amounts to, with regard to RT as a whole. The mechanism I have been describing—based on bottom-up activation of schemata, top-down activation of contextual information, and an assessment of these hypotheses on context against each other—is not intended to be an entirely alternative view of utterance interpretation. As I said, there are components of RT that I am explicitly endorsing, and others for which my proposal can be seen as an implementation from a neuro-computational perspective. In particular, I do not need to discuss the central core of the theory, that is, its rational reconstructions of the inferential structure leading from explicit meaning and a number of contextual assumptions to contextual implications. My proposal can rather be seen as a contribution to the understanding of such inferential mechanism, specifically, of how it is implemented by the basic activation/inhibition dynamic of the brain. My suggestion is that schemata at different levels of abstraction provide memory with the rational structure that is needed not only to activate explicit meaning and contextual assumptions (and implications), but also to assess which of these components of pragmatic inferences are coherent with each other and which are not.^8^

### How Goals Enter into the Picture

Now, I intend to argue that the above picture is entirely compatible with consideration of goals in utterance interpretation, in the line suggested by Paul Grice. Grice (1989) has described utterance understanding as a rational enterprise. More precisely, in his view the hearer assumes that the speaker is a rational agent pursuing her communicative goals and producing utterances that can be inferentially interpreted by the hearer as means to express those communicative intentions. Thus, in a sense utterance understanding is a matter of reconstructing coherent means-end structures. In this perspective, Grice also makes an appeal to context as a way to make guesses about the speaker’s goals, so as to license inferences backward to (what now is called) the explicit content of the utterance, as in the following example:

in cases where there is doubt, say, about which of two or more things an utterer intends to convey, we tend to refer to the context (linguistic or otherwise) of the utterance and ask which of the alternatives would be relevant to other things he is saying or doing, or which intention in a particular situation would fit in with some purpose he obviously has (e.g., a man who calls for a “pump” at a fire would not want a bicycle pump; Grice, 1957, p. 387).

In this example, since the context suggests the non-communicative goal of extinguishing a fire, the interpretation of a request for “a pump” is adjusted accordingly. A first thing to be stressed is the structural similarity with our previous example (2), where Peter’s question can be said to play the same role played here by extra-linguistic context: it settles the goal thanks to which the explicit meaning of Mary’s answer is adjusted (via a backward inference). That is, based on our knowledge of language we expect that Mary will adopt the goal of answering affirmatively or negatively Peter’s question. Assuming she has that goal, Mary can be expected to provide an explicit content which is a proper means to pursue it.

But not only is there a structural similarity which allows us to describe both RT’s and Grice’s examples in terms of the retrodiction of means from contextually inferred goals. Moreover, with regard to Grice’s example, it is natural to think that the man who calls for a “pump” has literally—not just as a figure of speech—the goal of extinguishing a fire. Having goals/intentions is legitimately considered constitutive of the notion of (intentional) action. If that is correct, in Grice’s example the representation of an extra-linguistic goal is key to the pragmatic interpretation of the man’s request. To the extent that this can be generalized, it seems that pragmatic processing needs to be embedded within a more general ability of mind-reading. This is explicitly recognized by Sperber and Wilson (2002), who mention approvingly Grice for having described human communication as a case of expression and recognition of intentions, define pragmatic interpretation as “an exercise in mind-reading” (Sperber and Wilson, 2002, p. 3), and propose in fact that the relevance-guided comprehension procedure is a “sub-module of the human mind-reading ability” (idem: 21). Although Sperber and Wilson do not draw such a conclusion, it seems reasonable to conclude that communicative intentions are embedded within wider goal structures and that this has a role to play in linguistic production and comprehension.^9^

Levinson (1992) has interestingly developed this idea in terms of the notion of “activity type.” Activity types are defined as social patterns of goal-directed behaviors in specific settings, delivering as such expectations about what’s going on next. Specifically, activity types raise expectations about the communicative actions to come. This means that communicative actions tend to be interpreted as moves in the current activity type, and therefore as something whose goals are expected to be sub-goals of the general activity. Levinson gives the following example: the sentence “C’mon Peter” may have a variety of meanings, but if one hears it during a basketball game it acquires a very clear sense, based on the kind of goal the speaker may have in that precise context. Other examples of activity types are trials and lessons, analyzed by Levinson in order to show that questions in English may have very specific uses (i.e., goals), which “are closely tied—indeed, derived from—the overall goals of the activities in which they occur” (idem: 82).

Let me summarize. Up to this point I have explored, mostly through an analysis of RT, the idea that utterance understanding is accomplished by a mechanism based on ease of access and the structure of memory. The key idea is that schemata in memory are activated (bottom-up) by multiple sources and then compete with each other for the (top-down) construction of cognitive contexts. I have also proposed that this process involves representation of goals.

In the rest of the paper, my purpose is to make this proposal both clearer and wider in scope by showing that a mechanism of the same sort has been invoked in a number of different cognitive domains.

## Schemata and Top-down Processes in the Cortex

### Concepts

As noted by relevance theorists, theories of memory assume that concepts are not isolated entities; they are organized instead in networks where some connections are stronger than others. Specifically, concepts are organized in chunks as a consequence of regular covariations, so as to ensure probabilistic coherence between them. For one example, Barsalou (2005) has argued for the notion of situated conceptualization, that is, the idea that conceptual representations in memory preserve information about specific settings in which the represented objects appear. On this background, Barsalou provides a nice formulation of the dynamic of activation between concepts and the situated conceptualizations they are embedded in:

The situated conceptualization that becomes active constitutes a rich source of inference. The conceptualization is essentially a pattern, namely, a complex configuration of multimodal components that represent the situation. When a component of this pattern matched the situation, the larger pattern became active in memory. The remaining pattern components-not yet observed-constitute inferences, that is, educated guesses about what might occur next. Because the remaining components co-occurred frequently with the perceived components in previous situations, inferring the remaining components is justified (Barsalou, 2005, p. 628).

It is easy to see that “patterns” are assigned here the same role played by schemata in our previous explanation of utterance understanding: a pattern or schema receives activation from any of its components and, once activated, it raises in turn the accessibility of its other components. Importantly, Barsalou’s analysis is not concerned with utterance understanding, it is devoted instead to explain the general functioning of concepts, specifically with regard to helping construct perception, predicting entities and events, supporting categorization, and providing inferences in general (idem: 621). Thus, it seems that our above explanation of utterance understanding is just a special case of a cognitive mechanism with a much wider scope.

### Language

As a matter of fact, a very similar mechanism is invoked by Ray Jackendoff (2007a) in his proposal of a parallel architecture in language processing. The main idea is that the generative engine at work in language production and comprehension is not exclusively based on syntax. On the contrary, syntax is just one of the layers involved—thanks to their respective principles of organization—in the generative arrangement of linguistic materials. Crucially, Jackendoff abandons the assumption of a radical distinction between grammar and lexicon, which was based on the idea that while lexicon is constituted by *representations*, syntactic rules are implemented instead by specific *processes*, with the former being inert entities processed by the latter. His alternative proposal is that linguistic entities at any layer, including syntactic structures, are bits of information stored in long-term memory and organized hierarchically, with higher levels prescribing the way in which items at lower levels must be arranged together. For each layer (syntax, semantics, phonology), the very same process of “unification” is held to be responsible for assembling specific items in accordance with the respective hierarchical organizations. Interestingly, Jackendoff’s proposal is just the most prominent representative of a general trend within syntactic theory, of which even Chomskyan minimalism is an example: that is, the trend toward the substitution of representations for procedural rules. In other words, the weight of explanation for language processing is nowadays mostly placed upon the organization of (linguistic) memory, not upon specialized processes.

On this background, Jackendoff describes the syntactic arrangement of a sentence as the result of a double movement: on the one hand, an initial word sets up “grammatical expectations” about the possible sentence structures, based on the syntactic patterns associated to that word at higher levels of the hierarchy; then, “further words in the sentence may be attached on the basis of the [previously activated] top-down structure” (Jackendoff, 2007a, p. 8). This amounts to the dynamic of bottom-up activation of schemata and top-down activation of their other components that is by now familiar to us. It is not a surprise, then, that Jackendoff characterizes the process as non-directional, such that it may work “from the bottom up or from top down or from anywhere in the middle” (idem: 8), and as based on competition between (and mutual inhibition of) alternative hypotheses, as in our previous description of pragmatic processing.

### Hierarchies in Action

Jackendoff’s theory of parallel architecture shows very convincingly how, as far as language is concerned, hierarchical organization of representations is apt to explain generative processing. But hierarchical representations have been taken to explain the generative nature of action as well.

The similarity between language and action with regard to their common generative nature is explicitly addressed in Jackendoff (2007b) and is largely recognized in psychological and neuroscientific theories of action (see Mazzone, 2014b, for a review). For one example, Baars and Gage (2010) observe that making plans for the future requires the ability to reconfigure elements of prior experiences in a way that does not exactly copy past experiences. This ability, they claim, is apparent in tool-making, one of the fundamental features of primate cognition, but “the generative power of language to create new ideas depends on this ability as well” (Baars and Gage, 2010, p. 402). According to the authors, “the ability to manipulate and recombine internal representations depends critically on the PFC [prefrontal cortex], which probably made it critical for the development of language” (idem: 402). We will turn below to this suggestion about PFC.

There is much research, in particular, on the relationship between hierarchical representations and generative processing in action understanding. Baldwin and Baird (2001, p. 171), for instance, claim that a “generative knowledge system underlies our skill at discerning intentions, enabling us to comprehend intentions even when action is novel and unfolds in complex ways over time” and suggest that this system “is probably just as rich and complex as the generative system underlying language” (idem: 171). They cite evidence that children can parse continuous actions along intention boundaries. However, they claim, the ability to parse and process hierarchically organized actions applies more generally:

Adults also appear to process continuous action streams in terms of hierarchical relations that link smaller-level intentions (e.g., in a kitchen cleaning-up scenario: intending to grasp a dish, turn on the water, pass the dish under the water) with intentions at higher levels (intending to wash a dish or clean a kitchen; Baldwin and Baird, 2001, p. 172).

The idea of a strict analogy (together with common neurological bases) between hierarchical structures in language and action is further developed by Pastra and Aloimonos (2012), which offer some detailed examples of how actions can be analyzed in terms of parse trees, within the framework of “a biologically inspired generative grammar of action, which employs the structure-building operations and principles of Chomsky’s Minimalist Program as a reference model” (Pastra and Aloimonos, 2012, p. 103).

Moreover, Glenberg and Gallese (2012) show how a mechanism that is firmly grounded in the study of motor control might have “been exploited for language learning, comprehension and production” (idem: 905). Their proposal is based on HMOSAIC (Haruno et al., 2003), which is a hierarchical version of MOSAIC, a model-based theory of motor control developed by Wolpert et al. (2003). Haruno et al. (2003) have demonstrated that, within such a hierarchical architecture, higher-level layers “can learn to select the basic motor acts and learn the appropriate temporal orderings of those acts” (Glenberg and Gallese, 2012, p. 910). The whole mechanism is explicitly described as associative, but the hierarchical structure allows nonetheless for abstract representations, standing as a whole for intentions of the agent: in practice, while at the lowest level in the model motor acts are simply chained with each other so that any of them triggers the next one, higher-level representations provide abstract patterns that capture action structure and timing more explicitly.

Let me summarize. In all of these approaches to action, flexible and generative processing is explained by means of hierarchical representations, in which patterns at higher levels prescribe predictable arrangements at lower levels. As it should be clear, those accounts place the explanatory weight on the organization of memory, not on specialized processes; in some case, simple associative processing is explicitly mentioned as the appropriate mechanism for memory acquisition and exploitation. This picture is entirely compatible with the above considerations on concepts and language processing, and with our previous account of pragmatic understanding. On the other hand, as we saw, consideration of action brings into focus notions such as goal and intention. It is therefore opportune to analyze in some detail how these notions are related to our key notion of schema.

### Schemata and Goals

It is reasonable to think that goals and intentions are complex entities, whose representation involves a number of components of different nature.^10^ However, for our purposes we can confine our attention to a simplified notion of goal/intention, along the lines of the above considerations on action. The idea—implicit in Baldwin and Baird (2001), Pastra and Aloimonos (2012), and Glenberg and Gallese (2012)—is that the goal underlying an action is the end-point of that action, with more complex actions being constituted by a sequence of smaller actions each of which is a means to (and a sub-goal of) the overarching goal, while actions at the bottom of the hierarchy are constituted by simple motor acts.

There are two points to this idea. The first concerns the existence of goal-directed patterns in memory, the second the thesis of a hierarchical structure of goals in the cortex.

As to the first point, Glenberg and Gallese (2012) argue, as we saw, that higher layers in HMOSAIC contain abstract patterns capturing the structure of actions. Based on our previous definition of schemata as the higher-level representations responsible for the organization of items at lower levels, such patterns can be legitimately considered as schemata. In the psychological, computational and neuroscientific literature on action, the existence of goal-directed patterns of this sort is commonplace. The most explicit defense of this claim—actually expressed in terms of the existence of “hierarchical schemas and goals in the control of sequential behavior”^11^—is provided by Cooper and Shallice (2006), mostly on the basis of computational considerations.^12^ They adopt the notion of schema proposed by Bartlett (1932) and further developed by Rumelhart and Ortony (1977) among others, according to which a schema is a self-contained memory structure with a variable number of component parts. In their words, as far as action control is concerned,

a schema may be seen as a means of achieving a goal or subgoal. More generally, recent computational accounts […] take schemas to be goal-directed structures, with goals serving to mediate schema–subschema relationships. Thus, schemas achieve goals and, apart from at the lowest level of the schema hierarchy, consist of partially ordered sets of subgoals (which may themselves be achieved by other schemas; Cooper and Shallice, 2006, p. 888).

Consistently, the authors describe the role that schemata play in action control in terms of the bottom-up/top-down dynamic we considered above: “Schemas are explicit and play a causal role in determining behavior: Excitation and subsequent selection of a schema cause excitation and then selection of subschemas or actions” (idem: 892).

The hierarchical organization of schema and goal representations is claimed to account for flexibility of sequential behavior (idem: 887)—an issue to which I will return in a moment. However, contextual flexibility is also explained by Cooper and Shallice by appealing to optional elements in schema representations. This would allow schemata to be highly context-sensitive, since optional subgoals can either be activated or not on any particular occasion as a function of the context in which the schema is performed (idem: 897). In order for this to be possible, schemata should also contain representations of the contextual cues whose excitation causes the activation of optional subgoals. The representation of contexts is explicitly mentioned by Badre (2013) as a component of what, in the literature on reinforcement learning of actions, is called a “policy,” that is, a rule that relates an action, a desired outcome and *a state in which the rule has to be applied*. This notion of context is clearly more specific than the one involved in our previous suggestion that schemata provides hypotheses about context. The point here is the specific requirement that certain situational cues must be present in order for certain goals to be pursued. Based on these considerations, we can describe a goal-directed schema as constituted by a final goal, a number of subgoals (or actions that are means to that goal), and some specification of the conditions in which both the final goal and the subgoals apply. In another, wider sense goal-directed schemata, as schemata in general, are chunks in memory providing appropriate contexts for each of their components.

The other important point, in the view of action as driven by hierarchies of goals, concerns the question whether such hierarchies are actually present in the brain, an issue that is better addressed on the background of a general understanding of brain architecture.^13^

### The Architecture of the Brain and the Prefrontal Cortex

As recently recalled by Badre (2013), Fuster (2001, 2003, 2014) was the first to associate a concept of abstraction in action control with the functional organization of frontal cortex. There is today some evidence that the hierarchical structure of goal-directed motor actions correlates with specific neurological regions (Hamilton and Grafton, 2006; Koechlin and Jubault, 2006; Grafton and Hamilton, 2007; Koechlin and Summerfield, 2007; Badre, 2008, 2013; Botvinick, 2008; Botvinick et al., 2009; O’Reilly, 2010). This suggests, in Botvinick’s (2008, p. 205) words, that “a topographical organization might exist within the frontal cortex and the DLPFC [dorsolateral prefrontal cortex], according to which progressively higher levels of behavioral structure are represented as one moves rostrally.” For one example of these studies, Koechlin and Jubault (2006, p. 936) reports evidence from magnetic resonance imaging showing “phasic activation at the boundaries of action segments that constitutes a hierarchical action plan”; on this basis, they propose that Broca’s area and its homolog in the right hemisphere might “implement a specialized executive system governing action selection in hierarchically structured action plans.”

Although the focus of those studies is on hierarchical representations of action in the frontal/prefrontal cortex, it should be noticed that on Fuster’s account hierarchical organization is a general phenomenon concerning the entire brain:

The physiology of the cerebral cortex is organized in hierarchical manner. At the bottom of the cortical organization, sensory and motor areas support specific sensory and motor functions. Progressively higher areas—of later phylogenetic and ontogenetic development—support functions that are progressively more integrative. The prefrontal cortex constitutes the highest level of the cortical hierarchy dedicated to the representation and execution of actions (Fuster, 2001, p. 319).

In other words, Fuster proposes that the brain is organized along two distinct—though highly interconnected—pathways, respectively constituting a sensory and a motor hierarchy of cortical maps, which together form a perception-action cycle. The PFC lies at the top of the motor hierarchy and it seems to contain neuronal networks that, both in monkeys and in humans, represent abstract programs or plans of action (Fuster, 2003, p. 76).

Two considerations are worth noting.

First, the above literature on action control emphasizes the role that hierarchies may play in flexibly dealing with large spaces of options. As Badre (2013) specifically notes, hierarchies permit a divide-and-conquer approach such that, on the one hand, choices about which actions to take can be made at multiple levels of abstraction, while, on the other hand, choices at the higher levels constrain the space of possible actions at lower levels. Compare this with a situation in which an inflexible routine has to be performed, and a single set of criteria for its application has to be coded and then assessed against the factual context. On the contrary, on the hierarchical account each component at lower levels has its own set of application criteria, and the selection of goals at the higher levels is the result of parallel activation of (and competition between) components at lower levels, with substantial gain in contextual flexibility. But this applies not only to goal selection in the frontal cortex: if Fuster—and our whole picture of the functioning of concepts, language and motor control—is right, the mechanism of bottom-up/top-down activations along hierarchical representations extends to the entire cortex, thus accounting for contextual flexibility in a wide range of cognitive processes.

Second, since we described the prefrontal cortex as the seat of hierarchical representations, one might wonder whether this is compatible with the well-established view according to which this area has a crucial role to play in executive processes. As a matter of fact, a “representational” versus “processing” approach to PFC has gained consensus in the last decade (Huey et al., 2006; Miller et al., 2002; Wood and Grafman, 2003), in line with the influential model of executive functions proposed by Miller and Cohen (2001). As they observe, “one of the most fundamental aspects of cognitive control and goal-directed behavior [is] the ability to select a weaker, task-relevant response (or source of information) in the face of competition from an otherwise stronger, but task-irrelevant one” (Miller and Cohen, 2001, p. 170). Now, Miller and Cohen’s suggestion is that the PFC contains patterns of activity which map onto configurations of representations in more posterior cortical areas. When such a pattern within the PFC is activated, this increases the activation of the posterior configuration it is connected to and allows that configuration to overcome task-irrelevant competing ones. In other words, plans of action in the PFC are here conceived as schemata, whose activation is transmitted to their components distributed in different cortical areas. This does not necessarily mean that the spreading of activation up and down the hierarchy is all there is to executive functions. An influential proposal made by Dehaene et al. (2006) is that self-sustaining loops play a crucial role in the neural dynamic, to the extent that they prevent the rapid decaying of spreading activation; more specifically, Dehaene et al. (2006) claim that consciousness depends on the establishing of such loops between strongly activated sensory-motor representations and higher association cortices. This might explain how prefrontal activation ensures stability of processing in accordance with current goals and tasks of the agent: thanks to recurrent loops, plans of action within the PFC might sustain the activation of related sensory-motor representations for the time needed to attain the goals. Under this account, there is no inconsistency between the suggestion that the PFC is the top of the hierarchy of representations in the cortex and the widespread opinion that it is key to conscious processing.

Executive functions are a third sense in which processes are usually said to be top-down. First, low-level processing can be constrained by higher-level schemata of various kinds; second, it can be specifically driven by plans of action, that is, by goal-directed schemata lying at the top of the perception-action cycle; third, it can be under the control of action plans in circumstances in which those plans and sensory-motor representations form self-sustaining loops. I claimed above that pragmatic processing is affected by top-down processing in the first two senses: in utterance interpretation, hypotheses about the cognitive context are constructed by exploiting the schematic organization of memory and, specifically, by activating goals within which the current communicative intention is embedded. I would like to suggest, though only in passing, that top-down processing in the third sense might have a role to play in utterance understanding as well. For instance, since in the normal case the speaker is consciously attended by the addressee, speaker-related information is likely to receive prominent activation in the course of utterance understanding (with consequences that are analyzed at some length in Mazzone, 2013).

## Conclusion

The main thesis defended in this paper is that, in understanding an utterance, the organization of memory is what essentially drives the construction of the appropriate cognitive context. More specifically, in the present account contextual assumptions and conclusions are provided by schemata, which are activated associatively by a variety of inputs (the utterance, its linguistic and situational context) and then assessed against each other. Goals have a crucial role to play in this process, insofar as goal-directed schemata are the highest levels in our cortical hierarchy of representations. I showed that this picture is consistent with suggestions made by RT and by Recanati, and with influential accounts of concepts, language, and action control. I also provided reasons to think that this hierarchical organization of memory and the related mechanism of bottom-up/top-down activation can account for generative processing and contextual flexibility.

The relation between the present account and RT invites some final comments. As I said above, despite the suggestions developed here, in its most general formulations RT takes a different view, based on expectations about the amount of cognitive effects and assessment of their actual amount against those expectations (in what follows, I will call this the “standard view”). In particular, let us focus on the fact that goal understanding plays no explicit role in this view. It is interesting to consider what Sperber and Wilson (1987) have to say on this issue:

Some commentators […] think our definition of relevance fails to do justice to pretheoretical intuitions. Utterances are relevant, they feel, to purposes, goals, topics, questions, interests, or matters in hand.

We define relevance in a context and to an individual. We say what a context is, how it is constructed and how, once constructed, it affects cognition and comprehension. One reason we did not set out to define relevance to a purpose, goal, and so on, is that we had no idea how to answer the analogous questions for any of these terms […]. Given a definition of relevance in a context, and a method of context construction, however, there is no reason that assumptions about the goals and purposes of the individual, or of the participants in a conversation, should not form part of the context and give rise to contextual effects in the usual way. Such assumptions are likely to be particularly rich in contextual effects, since purposes and goals imply plans for action. We see no incompatibility between our work and a belief in the importance of goals, purposes, and plans; on the contrary, RT sheds light on how these important notions may play the roles they play (Sperber and Wilson, 1987, p. 742).

The suggestion is that explaining comprehension directly in terms of goals is at least a difficult (and perhaps an impossible) enterprise. Most of all, according to the authors such explanation is not needed anyway, since RT can account for the importance of goals in comprehension without any explicit mention of them. However, the standard view might succeed in this ambition only by providing a satisfying account of how the amount of cognitive effects is assessed, while in fact we are left with no better explanation of this than the speculation about “symptomatic physico-chemical changes.” On the other hand, we provided here at least the general sketch of an explanation of comprehension based on schemata and goals, which is in fact consistent with the following ideas of RT: the interpretation process requires the construction of an appropriate cognitive context; this depends on the organization of memory, which determines the ease of access of contextual assumptions and conclusions; a mutual adjustment occurs between explicit meaning, contextual assumptions and contextual conclusions; specifically, backward inferences are based on expectations about the type of intended cognitive effects. The account of comprehension developed in this paper along those lines appears better grounded than the standard view, if only for the following two reasons.

First, it makes an appeal not to controversial claims about sensitivity to cognitive costs and effects, but instead to well-established cognitive facts (mechanisms of associative access and the organization of memory in chunks), which can be argued to play a key role in theories of concepts, language, and action control, and specifically in the explanation of contextual flexibility in those domains.

Second, this account embeds utterance understanding within a general ability to understand goals, in line with Grice’s view and in accordance with explicit claims made by Sperber and Wilson. Interestingly, Sperber and Wilson’s notion of relevance is, in a sense, a reinterpretation of Grice’s maxim of quantity. There is, though, a clear difference between the two as to how they conceive the purpose of communication: while the former assumes that the speaker aims to be as informative as possible (compatibly with considerations of effort), the maxim of quantity prescribes instead that the speaker is “as informative as is required (*for the current purposes* of the exchange)” (Grice, 1975, p. 45; emphasis mine). In other words, in Grice’s account the amount of information exchanged is not a purpose in itself; it is instead a means for pursuing other goals. From this point of view, the notion of relevance proposed by Sperber and Wilson seems to fall back into a pre-pragmatic view in which communication is conceived as instrumental not to the variety of human actions and goals, but instead to the acquisition and transmission of knowledge *per se*. On the contrary, in line with Fuster’s proposal of a perception-action cycle, the view defended here is that communication in particular, as well as cognition in general, is geared to goal management and action (instead of to maximization of information). This makes communication, in Tomasello’s (2008, p. 49) words, an exercise in “practical reasoning.”

It is, I maintain, RT’s notion of relevance that is in the end responsible for the problems affecting the standard view. The point is that it is very difficult (and perhaps impossible) to give a sensible cognitive instantiation to the idea of maximization of information. If we abandon this idea, even the above quotation may make new sense. As Sperber and Wilson say, “there is no reason that assumptions about the goals and purposes of the individual […] should not form part of the context and give rise to contextual effects.” In fact, I maintain, goal representations are part of our repertoire of schemata in memory and they can contribute to determine context via backward inferences. But this is because communication is essentially a goal-oriented activity.

In sum, my claim is that the quantitative notion of relevance and the related idea of a quantitative assessment of cognitive benefits raise serious problems. In my view, the good news for RT is that large parts of the theory stay unaffected by these problems.

### Conflict of Interest Statement

The author declares that the research was conducted in the absence of any commercial or financial relationships that could be construed as a potential conflict of interest.

## Footnotes

1 Strictly speaking, the inference is from the explicit meaning to the intended conclusions, with explicit meaning being the result of pragmatic processes applied to coded meaning. However, for the sake of simplicity I will speak of coded meaning whenever the distinction is of no import to our discussion.

2 Let me introduce explicit definitions of the most important terms I will use. I call “schema” any higher-level cognitive representation, which is apt to specify the relationships between its components at a lower level. Schemata are based on co-occurrences in previous experience and they provide memory with structure. In the present context, I mainly use “bottom-up activation” to refer to the process by which pieces of information activate the schemata they pertain to, while “top-down activation” is the process by which the activation of a schema activates in turn its (other) components (for a different sense of “top-down,” see below The Architecture of the Brain and the Prefrontal Cortex). “Competition” among representations, and specifically among schemata, may occur for the simple fact that they are differentially activated and, therefore, one has stronger effects than the other. However, strictly speaking, activation is just one side of the coin: there can be both excitatory and inhibitory links between representations. As a consequence, competition can also occur by way of inhibition, when schemata represent alternative state of affairs. For an example, see below (example 1) the discussion of how the ambiguity between two meanings of “bank” is resolved thanks to the activation of a schema for GETTING MONEY FROM THE BANK1. Although I will emphasise the excitatory role of this schema on the contextually appropriate meaning, it should also be considered the possibility of an inhibitory link between this meaning and its alternative(s).

3 With the further qualification that my proposal has consequences whose compatibility with other aspects of RT requires discussion: for a first example, RT seems to be committed to the view that the inferential component of comprehension cannot be implemented in associative terms (for a discussion, see Mazzone, 2014a); for a second example, my proposal seems to trivialize the notion of modularity (Mazzone, submitted) in a way that might not fit with relevance theorists’ views.

4 But see note 1.

5 Recanati prefers the plural “schemata” whereas Sperber and Wilson use “schemas.” From now on, I will always use the former for the sake of uniformity. For a more extensive discussion of the notion of schema, see Mazzone (2014a).

6 Mazzone (2011a, 2014a) argues for a generalization of this explanation (based on associative processing and schemata) beyond the limits of “primary pragmatic processes.”

7 As correctly pointed out by one of the referees, relevance theorists have developed a view of lexical pragmatics (with an important role for the notion of ad hoc concepts) that is not mainly based on ease of access. This view is in fact consistent with their general assumption that, although associative links may affect the accessibility of contextual assumptions and conclusions, the overall interpretation will only be accepted “if it satisfies the hearer’s expectations of relevance and is properly warranted by the inferential comprehension heuristic” Wilson and Carston (2006, p. 429). I have discussed these proposals in more details elsewhere (for RT’s lexical pragmatics, see Mazzone, 2011a, 2014c; for ad hoc concepts, Mazzone, 2014a). My only point here is that, insofar as RT’s lexical pragmatics ultimately depends on the inferential comprehension heuristic and expectations of relevance, it is crucial to understand how those expectations are assessed. In section context and relevance I raised a problem for the standard RT’s proposal based on the quantitative notion of expectations of relevance, while in the next section I argue that that problem can be avoided by adopting a different, qualitative, notion.

8 For a wider discussion of this idea, see Mazzone (2014a).

9 This proposal is further analysed in Mazzone (in press). One of the referees observes that RT has developed a complex account of the role of mindreading, metarepresentations and the mechanism of epistemic vigilance in utterance understanding. Although there is no room to address here in any detail the issue, my view might be intended as a proposal about the low-level implementation of mindreading (an associative account of mindreading is defended in Mazzone, 2014d).

10 Mazzone (2011b) proposes that goals can be analysed in terms of (a) motoric and perceptual representations of end-states; (b) attributions of value to those representations by the reward system; (c) representation of means to those ends together with appropriate contexts (including an appreciation of the fact that, for a given end-state, different means are needed in different contexts). Moreover, intentions are usually thought of as consciously attended goals.

11 This is in fact the title of the paper.

12 The defence of goal-directed schemata in Cooper and Shallice (2006) is part of a larger debate, markedly with Botvinick and Plaut, about symbolic and connectionist models of action representation. Interestingly, in their reply to Cooper and Shallice (2006); Botvinick and Plaut (2006) admit that schemata and goals need to be represented somehow, they only object that “it is too strong to say [that their own model] is eliminativist with respect to task and subtask representations (i.e., schemas), it is true that the relevant patterns of activation may be more difficult to isolate within [their model than in the one proposed by Cooper and Shallice]” (Botvinick and Plaut, 2006, p. 921). Moreover, they argue for a “quasi-hierarchical structure” of action representation (idem: 922), that is, a structure in which there is a balance between hierarchy and context sensitivity—I will say in a moment something more on context sensitivity in hierarchical representations. In sum, none of the claims we report here from Cooper and Shallice (2006) is really disputed by Botvinick and Plaut (2006).

13 One of the referees has correctly pointed out that there is neuroscientific literature on pragmatic processing and the interplay between pragmatics and intention recognition—involving different areas than the PFC—which is not accounted for in this paper (see, for instance, Catani and Bambini, 2014; Hagoort and Levinson, 2014). However, I want to emphasize that the purpose of the next section is not to address the neuroscience of pragmatics; it is instead to show that also neuroscience has proposed a hierarchical organization of representations, in line with cognitive theories of concepts, language, and action. This is further support to my general view of context construction as based on a bottom-up/top-down dynamic of activation in hierarchical representations. Thus, what I am interested in is theorizing (together with the supporting evidence) about hierarchical representations in the brain. The PFC is especially well studied in this regard, in particular in connection with the issue of goal representation, and this is why I focus my attention on it. This said, the issue of how the PFC and other cortical areas contribute to the representation of intentions and goals undoubtedly requires further investigation.
